# Medication adherence and costs of medical care among patients with Parkinson’s disease: an observational study using electronic medical records

**DOI:** 10.1186/s12889-024-18431-y

**Published:** 2024-04-30

**Authors:** Zhanmiao Yi, Yudan Mao, Chenxuan He, Yantao Zhang, Junwen Zhou, Xing Lin Feng

**Affiliations:** 1 Department of Pharmacy, Peking University Third Hospital, 49 North Garden Road, Haidian District, 100191 Beijing, China; 2 Institute for Drug Evaluation, Peking University Health Science Center, Beijing, China; 3 Department of Pharmacy, Hospital of Renmin University of China, Renmin University of China, Beijing, China; 4 Institute of Statistics and Big Data, Renmin University of China, Beijing, China; 5 State Grid Digital Technology Holding Co., LTD, Beijing, China; 6 Health Economics Research Centre, Nuffield Department of Population Health, University of Oxford, Oxford, UK; 7 School of Public Health, Peking University, Haidian District, 100191 Beijing, China

**Keywords:** Medication adherence, Direct medical cost, Medication possession ratio, Parkinson's disease, Proportion of days covered

## Abstract

**Background:**

Adherence to antiparkinsonian drugs (APDs) is critical for patients with Parkinson’s disease (PD), for which medication is the main therapeutic strategy. Previous studies have focused on specific disorders in a single system when assessing clinical factors affecting adherence to PD treatment, and no international comparative data are available on the medical costs for Chinese patients with PD. The present study aimed to evaluate medication adherence and its associated factors among Chinese patients with PD using a systematic approach and to explore the impact of adequate medication adherence on direct medical costs.

**Methods:**

A retrospective analysis was conducted using the electronic medical records of patients with PD from a medical center in China. Patients with a minimum of two APD prescriptions from January 1, 2016 to August 15, 2018 were included. Medication possession ratio (MPR) and proportion of days covered were used to measure APD adherence. Multiple linear regression analysis was used to identify factors affecting APD adherence. Gamma regression analysis was used to explore the impact of APD adherence on direct medical costs.

**Results:**

In total, 1,712 patients were included in the study, and the mean MPR was 0.68 (± 0.25). Increased number of APDs and all medications, and higher daily levodopa-equivalent doses resulted in higher MPR (mean difference [MD] = 0.04 [0.03–0.05]; MD = 0.02 [0.01–0.03]; MD = 0.03 [0.01–0.04], respectively); combined digestive system diseases, epilepsy, or older age resulted in lower MPR (MD = -0.06 [-0.09 to -0.03]; MD = -0.07 [-0.14 to -0.01]; MD = -0.02 [-0.03 to -0.01], respectively). Higher APD adherence resulted in higher direct medical costs, including APD and other outpatient costs. For a 0.3 increase in MPR, the two costs increased by $34.42 ($25.43–$43.41) and $14.63 ($4.86–$24.39) per year, respectively.

**Conclusions:**

APD adherence rate among Chinese patients with PD was moderate and related primarily to age, comorbidities, and healthcare costs. The factors should be considered when prescribing APDs.

**Supplementary Information:**

The online version contains supplementary material available at 10.1186/s12889-024-18431-y.

## Introduction

Parkinson’s disease (PD) is the second most prevalent neurodegenerative disease globally, affecting adults 65 and older [1]. With an aging population, the disability-adjusted life years for PD more than doubled globally between 1990 and 2016 [2]. As PD progresses, complications such as non-motor symptoms and gait problems increase [3], significantly affecting the patient’s quality of life [4]. It is estimated that the direct medical cost for PD is approximately $14.2 billion in the US, with $4.8 billion attributed to loss of income [5]. In China, it is estimated that there will be approximately five million patients with PD by 2030, accounting for almost 50% of the global PD population [6]. Meanwhile, drug costs accounted for 97.82% of outpatient medical expenditures [7]. To alleviate the economic burden on families with PD patients, PD has been included in major disease insurance in the Chinese government insurance system since 2007 [8].

Antiparkinsonian medication is an essential therapeutic strategy for patients with PD [9, 10]. Poor adherence to antiparkinsonian drugs (APDs) may not only reduce patients’ quality of life but also predispose them to complications associated with PD, thereby increasing mortality risk [11, 12]. Adequate APD adherence, however, can alleviate PD symptoms and motor complications, improve quality of life [13], and decrease all-cause healthcare costs by reducing ambulatory visits, admissions, and lengths of stay [14–16]. A recent review indicated that achieving target medication adherence would save €239,000–€576,000 in Germany and €917,000–€2,980,000 in the UK for every 1,000 patients over 1.5 years, respectively [17].

Previous studies on APD adherence have considered demographic and social factors (e.g., age, marital status, educational level, and income), as well as clinic-related factors, such as motor and non-motor symptoms, primarily cognitive and mood symptoms, disease duration, and regimen complexity [14, 18]. However, when evaluating the clinical factors that affect adherence to PD treatment, the literature has only focused on specific diseases within a single system, such as depression [16, 19] and gastrointestinal diseases [16]. However, managing the care and medications of patients is complex and requires multidisciplinary clinical expertise [20]. To improve patient benefits, a systematic approach is warranted to understand the effect of multiple comorbidities, including other neurological diseases and systems.

To the best of our knowledge, no international comparative data are available on the medical costs for Chinese patients with PD [7, 21]. Most studies have been based on survey data, which have limited accuracy with regard to cost estimates [7]. Using electronic medical records in a tertiary hospital in China, the present study reports medication adherence and medical costs for patients with PD. The two measures for assessing medication adherence were ascertained: medication possession ratio (MPR) and proportion of days covered (PDC). They focused on clinical factors that comprehensively included PD-related neurological diseases and disorders of other systems to systematically understand barriers to patient adherence to medication in real-world practice. Subsequently, the additional direct medical costs that are associated with medication adherence were estimated.

## Methods

### Ethical approval of study

This study was conducted in accordance with the guidelines of the Declaration of Helsinki (revised in 2013) and approved by the Institutional Ethics Board of Peking University Third Hospital (No. IRB00006761-M2018228).

### Data and procedures

Data on outpatient visits and inpatient admissions were extracted from the hospital information system of Peking University Third Hospital from January 1, 2016 to August 15, 2018. Peking University Third Hospital is a tertiary care medical center and teaching institution located in Beijing, China, which started the first collaborative pharmaceutical care service for patients with PD in 2017 [20]. Patients newly diagnosed with PD or those with a history of PD were included. Diagnoses were identified using the International Classification of Diseases, 10th Revision, Clinical Modification code (ICD-10 code: G20), and relevant terms (e.g., PD). Data on patient identifiers, age, sex, date of visit/discharge, type of health insurance, diagnosis, prescriptions, and charges were extracted from outpatient records, whereas data on date of admission, total prior admissions, and expenditures aggregated annually were extracted from inpatient records.

Claim data were used to ascertain the prescribed medications filled. The pharmacy at Peking University Third Hospital administered eight APDs covering six major classes: dopamine precursors (levodopa/benserazide and carbidopa/levodopa), dopamine agonists (pramipexole and piribedil), selective monoamine oxidase B inhibitors (selegiline), catechol-O-methyltransferase inhibitors (entacapone), amantadine, and trihexyphenidyl. A prescription containing any of the eight APDs was considered an APD prescription [7]. Patients with PD who were prescribed APDs at a minimum of two outpatient visits during the study period were included. For each outpatient visit, data on all relevant information related to APD prescriptions, including the generic and brand names, quantity, number, and cost of APDs, were retrieved. Each patient was indexed for the first visit (initiation date) with an APD prescription during the study period and followed up until the last visit when their prescribed volume of APDs was used.

### Outcomes

#### Adherence to medication

Two indicators were used to measure APD adherence: MPR and PDC [12, 22–24]. Both MPR and PDC indicate the percentage of days a patient had access to APDs throughout the “duration of treatment,” calculated for each patient as the number of days between the first and last APD prescription, inclusive of estimated days when last prescription of APDs would have been used. MPR and PDC have the same denominator but different numerators. The numerator of MPR is the average number of days of prescription coverage for each APD, whereas that of PDC is the total number of days that a patient had at least one prescription for any APD. The equations used to calculate MPR and PDC are shown in Fig. S1. In the univariate analysis, APD adherence was categorized into three groups: low, medium, and high, representing poor, partial, and good adherence, with 0.6 and 0.8 as the cut-off points for MPR and PDC, respectively [12, 25, 26].

#### Direct medical costs

Direct medical costs were estimated using a bottom-up approach, for which all charges from electronic medical records were summed and then the data was rescaled on an annual basis. Direct medical costs included APD costs (charges for any prescription of the eight APDs), non-APD costs, other outpatient visit costs (charges for consultation, test/examination, and other consumables), and inpatient admission costs (charges for diagnosis, examination, medication, nursing, and other consumables during hospitalization). Total medical costs were defined as the sum of all expenses for each patient based on the records of outpatient visits and inpatient admissions.

### Main explanatory variables

#### Concurrent neurological diseases

All concurrent neurological diseases for each patient with PD were included by screening the codes of the ICD-10, and Chinese keywords for diagnoses, and the following diagnoses were selected: depression, mental disorder, dementia, sleep disorder, and epilepsy.

Concurrent neurological diseases were selected as variables for the baseline status rather than the entire follow-up period, avoiding the impact of time and ensuring the predictability of study results. The baseline status was the patient’s condition at the beginning of the study, from 180 d before the initiation date to 90 d after.

#### Other comorbidities

The top 50 non-APDs were counted based on the frequency of outpatient prescriptions. Guided by the pharmacological classification and indications for the medications, several other systemic diseases (excluding neurological diseases) were defined as comorbidities. The search method was the same as that used for neurological diseases.

#### Other covariates

Age, sex, medical insurance, inpatient admission, baseline number of APDs, baseline number of all medications, and baseline concurrent diseases were included as variables. Additionally, the daily levodopa-equivalent dose (LED) at baseline was selected as a variable, which was derived by dividing the number of prescribed APD doses at baseline by the conversion factors of the LED [7]. Daily LED combined with the number of APDs may reflect PD severity at baseline to compensate for lack of PD stages in the data. Adherence indicators (MPR and PDC) were included as variables to investigate the medical costs of patients with PD.

### Statistical analysis

R programming language was used to analyze the data. Multiple linear regression analysis was conducted to investigate the factors associated with medication adherence and forest plots of MD were present. The data were adjusted for patient characteristics, including age, sex, baseline concurrent diseases, health insurance, baseline number of APDs, baseline number of all medications, baseline daily LED, and inpatient admissions. The backward method was used to systematically select factors and the standardized regression coefficients were reported. To investigate the factors associated with medical costs, generalized linear models were constructed using the identity link function and gamma distribution in the parameterization. The same stepwise methods were used to select covariates and distinct factors for different types of costs were ultimately identified. The tests were two-sided, and P values < 0.05 indicated statistical significance.

### Sensitivity analysis

Two types of sensitivity analyses were performed. While MPR is a measure commonly used to assess medication adherence, PDC is increasingly being applied [11, 18, 27]. Therefore, in the present study, the regression results for MPR are reported as the primary analysis, and those for PDC are reported as the sensitivity analysis. Second, to test whether the length of follow-up would affect the findings, the follow-up period was divided into three quantiles, that is, ≤ 290 d, 290–800 d, and ≥ 801 d.

## Results

### Patient characteristics

The electronic medical records of 2,640 patients with PD were retrieved from the Peking University Third Hospital. After excluding 214 patients who were not prescribed APDs and 714 patients who were prescribed APD only once, there were 1,712 patients in the present study (Fig. S2). Among the 1,712 patients, 976 were men (57.0%) and 736 were women (43.0%). The mean (standard deviation [SD]) age was 70.1 (11.8) years. The mean (SD) follow-up days were 538.97 (334.14) d. The mean (SD) number of different types of APDs used was 2.52 (1.51) per patient, whereas the mean (SD) number of different types of other medications was 6.23 (6.93). The mean (SD) baseline daily LED was 0.3853 (0.2725) g.

Based on the 50 non-APDs (Table S1) with the highest prescription frequency, the following target concurrent diseases were assessed: (1) circulatory system diseases, including hypertension and ischemic cardiomyopathy; (2) endocrine and metabolic diseases, including diabetes and lipoprotein metabolism disorders; and (3) digestive system diseases, including constipation and diseases of the esophagus, stomach, and duodenum. Out of all the patients with PD, 41.5% (*n* = 710) had concurrent circulatory system diseases, the largest proportion, and 20.9% (*n* = 358) were diagnosed with digestive system diseases (Table 1). The most common concurrent neurological disease was sleep disorder, followed by depression, dementia, and mental disorder.

**Table 1:** Characteristics and MPRs of the included patients with PD

Characteristics	Patients, n (%)	MPR, Mean (SD)
Total sample	1712 (100)	0.68 (0.25)
**Baseline**		
**Sex**		
Female	736 (43.0)	0.67 (0.25)
Male	976 (57.0)	0.69 (0.25)
**Age range, y**		
≤ 44	33 (1.9)	0.69 (0.26)
45–64	529 (30.9)	0.70 (0.26)
65–74	404 (23.6)	0.69 (0.24)
75–84	616 (36.0)	0.67 (0.25)
≥ 85	130 (7.6)	0.62 (0.25)
**Number of all medications**		
1	153 (8.9)	0.58 (0.28)
2–5	998 (58.3)	0.67 (0.25)
6–10	493 (28.8)	0.72 (0.24)
11–36	68 (4.0)	0.75 (0.23)
**Concurrent diseases**		
**Neurological diseases**		
Depression: Yes	313 (18.3)	0.69 (0.25)
Depression: No	1399 (81.7)	0.68 (0.25)
Mental disorders: Yes	195 (11.4)	0.69 (0.25)
Mental disorders: No	1517 (88.6)	0.68 (0.25)
Dementia: Yes	286 (16.7)	0.67 (0.25)
Dementia: No	1426 (83.3)	0.68 (0.25)
Epilepsy: Yes	58 (3.4)	0.60 (0.32)
Epilepsy: No	1654 (96.6)	0.68 (0.25)
Sleep disorders: Yes	378 (22.1)	0.69 (0.23)
Sleep disorders: No	1334 (77.9)	0.68 (0.26)
**Circulatory system diseases: Yes**	710 (41.5)	0.67 (0.25)
**Circulatory system diseases: No**	1002 (58.5)	0.69 (0.25)
**Endocrine and metabolic diseases: Yes**	600 (35.0)	0.67 (0.26)
**Endocrine and metabolic diseases: No**	1112 (65.0)	0.69 (0.25)
**Digestive system diseases: Yes**	358 (20.9)	0.66 (0.23)
**Digestive system diseases: No**	1354 (79.1)	0.69 (0.26)
**Medical insurance: Yes**	1114 (65.1)	0.68 (0.25)
**Medical insurance: No**	598 (34.9)	0.69 (0.26)
**Follow-up period**		
** Follow-up days, d**		
≤ 290	565 (33.0)	0.78 (0.24)
291–800	574 (33.5)	0.60 (0.26)
≥ 801	573 (33.5)	0.67 (0.23)
**Number of outpatient visits**		
2–6	815 (47.6)	0.67 (0.29)
7–12	383 (22.4)	0.63 (0.24)
13–18	235 (13.7)	0.65 (0.18)
19–24	137 (8.0)	0.77 (0.17)
≥ 25	142 (8.3)	0.86 (0.14)
**Inpatient episode: Yes**	115 (6.7)	0.68 (0.23)
**Inpatient episode: No**	1597 (93.3)	0.68 (0.25)
**Number of APDs**		
1	578 (33.8)	0.59 (0.29)
2	390 (22.8)	0.69 (0.25)
3	361 (21.1)	0.73 (0.21)
4	233 (13.6)	0.76 (0.20)
5–7	150 (8.8)	0.78 (0.16)
**Number of APD regimens**		
1	623 (36.4)	0.59 (0.29)
2	280 (16.4)	0.72 (0.24)
≥ 3	809 (47.3)	0.74 (0.21)
**Daily number of APDs**		
1	602 (35.2)	0.56 (0.28)
> 1 and ≤ 2	744 (43.5)	0.66 (0.24)
> 2 and ≤ 3	289 (16.9)	0.74 (0.21)
> 3 and ≤ 5	77 (4.5)	0.86 (0.14)

*Abbreviations* APD, antiparkinsonian drug; MPR, medication possession ratio; PD, Parkinson’s disease; SD, standard deviation

### Adherence to PD medication

The mean (SD) MPR of all patients was 0.68 (0.25) (Table 1). A total of 633 (37.0%), 424 (24.8%), and 655 (38.2%) patients were classified as having low, medium, and high medication adherence, respectively.

A total of 578 (33.8%) patients received antiparkinsonian monotherapy. The mean (SD) MPR and PDC were 0.59 (0.29) and 0.57 (0.28), respectively. A total of 447 (77.3%), 75 (13.0%), 15 (2.6%), and 20 (3.5%) patients received levodopa/benserazide, pramipexole, piribedil, and selegiline, respectively, and the remaining 21 (3.6%) received the other three APDs. The results are summarized in Table S2.

**Fig. 1:**
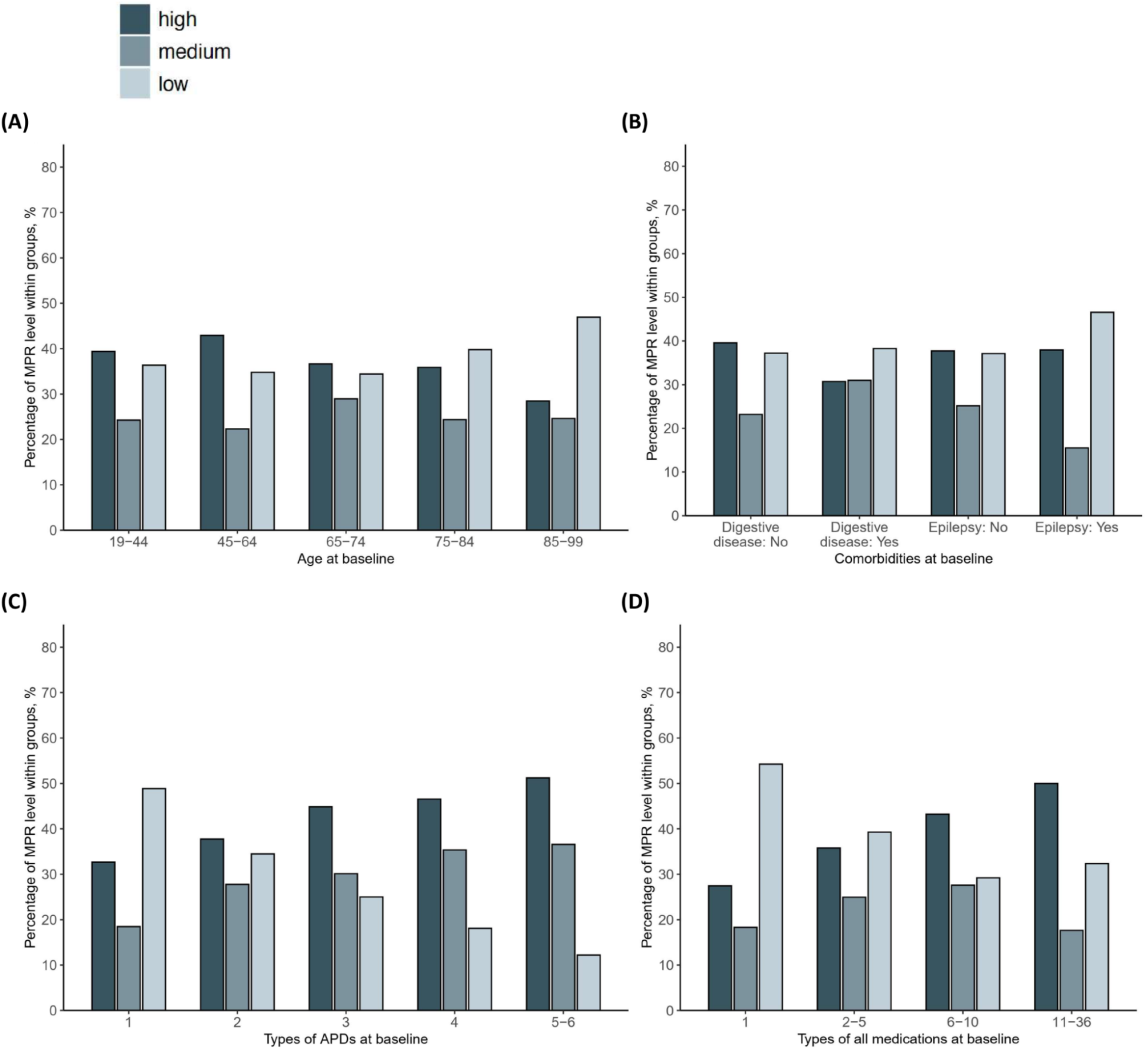
Factors associated with adherence and the percentage of patients with low, medium, and high MPR in each subgroup. (**A**) Percentage of patients with low, medium, and high MPR in different age groups. (**B**) Percentage of patients with low, medium, and high MPR in the subgroup with comorbid digestive system disease or epilepsy. (**C**) Percentage of patients with low, medium, and high MPR in the subgroup with different types of APDs. (**D**) Percentage of patients with low, medium, and high MPR in the subgroup with different types of all medications. Abbreviations: APD, antiparkinsonian drug; MPR, medication possession ratio

The results of univariate analysis suggest that the MPR may be related to patient age, prevalence of digestive system diseases, epilepsy, and the number of APDs and all medications (Fig. 1). For example, the proportion of patients aged > 45 years in the high-MPR group decreased gradually (Fig. 1A). Approximately 69.3% of patients with comorbid digestive system diseases had low or medium MPR. Among patients with comorbid epilepsy, 46.6% were in the low MPR group, compared to 37.1% of those without epilepsy (Fig. 1B). The proportion of patients with a high MPR increased gradually with an increase in the number of APDs and all medications used (Fig. 1C, D).

**Fig. 2:**
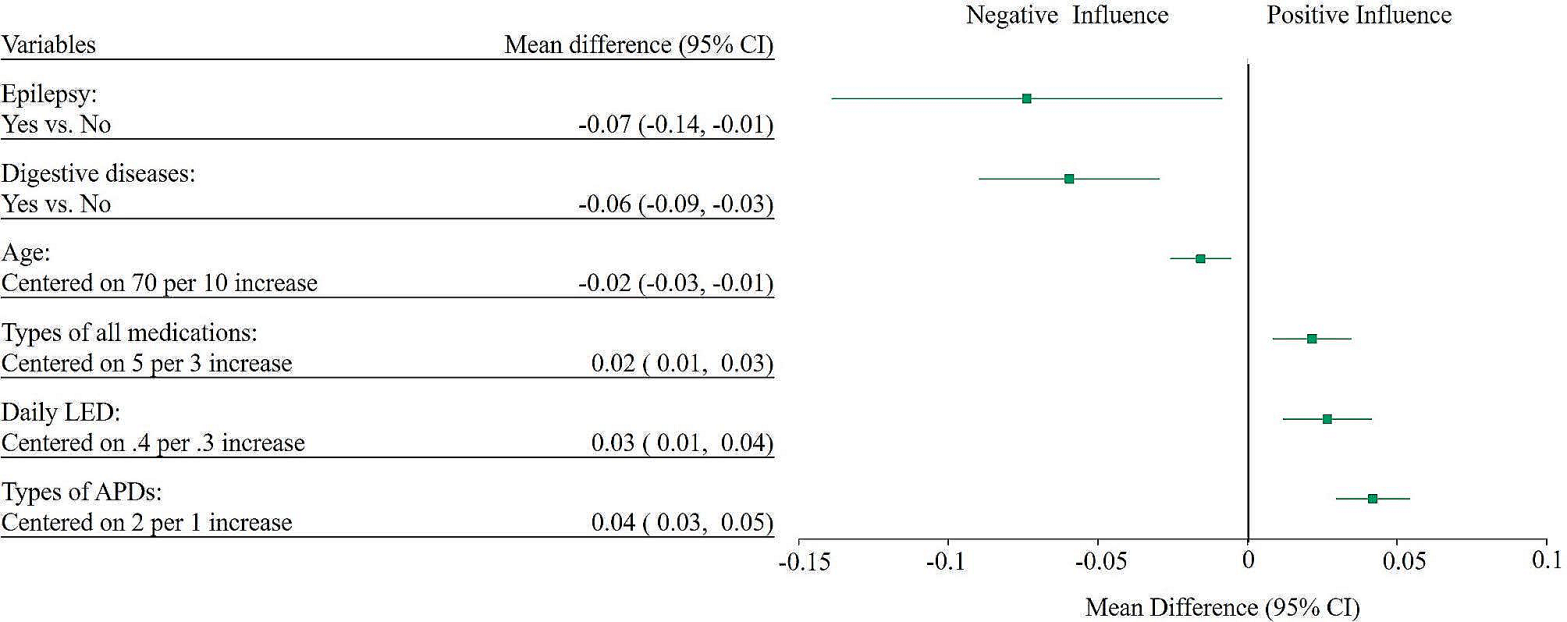
Multiple linear regression analysis for APD adherence^a^. ^a^Stepwise regression was performed. Using the backward method, insignificant variables were dropped step-by-step. Hence, significant variables with a p-value less than 0.05 were selected in the end. Abbreviations: APD, antiparkinsonian drug; CI, confidence interval; LED, levodopa-equivalent dose

The results of multivariate regression analyses for determinants of MPR indicated persistent effects of age (MD, -0.02; 95% confidence interval [CI], -0.03 to -0.01), concurrent digestive system diseases (MD, -0.06; 95% CI, -0.09 to -0.03), epilepsy (MD, -0.07; 95% CI, -0.14 to -0.01), number of APDs (MD, 0.04; 95% CI, 0.03 to 0.05), number of all medications (MD, 0.02; 95% CI, 0.01 to 0.03), and daily LED (MD, 0.03; 95% CI, 0.01 to 0.04) on APD adherence (Fig. 2).

### Medical costs associated with PD

The mean (SD) annual total medical costs, outpatient visit costs, and inpatient admission costs per patient were $1,604.56 ($1,520.27), $1,477.82 ($1,284.71), and $126.74 ($963.69), respectively. The mean (SD) annual cost for medications was $1,449.15 ($1,279.22), accounting for more than 98% of outpatient visit costs.

Table 2 illustrates the factors associated with medical costs. The Gamma regression analysis results showed that higher APD adherence rates resulted in higher direct medical costs, particularly APD costs. For an increase of 0.3 in MPR, APD costs increased by $34.42 (95% CI, $25.43–$43.41) per patient per year. Moreover, for every 10-year increase in age, the cost of APDs and non-APDs increased by $13.58 (95% CI, $6.93–$20.23) and $5.36 (95% CI, $4.41–$6.32) per patient per year, respectively.

**Table 2:** Gamma regression analysis of medical costs with MPR adherence measures^a^

Variables	APD costs			Non-APD costs			Other outpatient costs		
	Estimate	95% CI	*P value*	Estimate	95% CI	*P value*	Estimate	95% CI	*P value*
Intercept	526.28	(495.24, 557.32)	< 0.001	199.99	(171.65, 228.32)	< 0.001	112.84	(95.16, 130.53)	< 0.001
Age (centered on 70 per 10 increase)	13.58	(6.93, 20.23)	< 0.001	5.36	(4.41, 6.32)	< 0.001	-14.12	(-22.43, -5.82)	< 0.001
Sex (male vs. female)	ND^b^	ND	ND	8.15	(6.58, 9.71)	< 0.001	-17.24	(-33.91, -0.56)	0.04
Medical insurance (yes vs. no)	-26.31	(-50.18, -2.43)	0.03	3.87	(3.04, 4.70)	< 0.001	ND	ND	ND
MPR (centered on 0.7 per 0.3 increase)	34.42	(25.43, 43.41)	< 0.001	ND	ND	ND	14.63	(4.86, 24.39)	0.003
Number of APDs (centered on 2 per 1 increase)	327.13	(300.81, 353.46)	< 0.001	-64.24	(-73.71, -54.77)	< 0.001	ND	ND	ND
Number of medications (centered on 5 per 3 increase)	-28.08	(-36.00, -20.15)	< 0.001	183.68	(155.11, 212.26)	< 0.001	ND	ND	ND
Circulatory diseases (yes vs. no)	ND	ND	ND	46.15	(18.98, 73.32)	< 0.001	ND	ND	ND
Endocrine and metabolic diseases (yes vs. no)	-32.84	(-51.70, -13.97)	< 0.001	65.36	(28.50, 102.21)	< 0.001	ND	ND	ND
Digestive diseases (yes vs. no)	79.84	(49.27, 110.42)	< 0.001	ND	ND	ND	ND	ND	ND
Mental disorders (yes vs. no)	-29.89	(-49.40, -10.37)	0.003	ND	ND	ND	29.41	(0.84, 57.98)	0.04
Dementia (yes vs. no)	ND	ND	ND	499.4	(356.10, 642.70)	< 0.001	ND	ND	ND
Depression (yes vs. no)	ND	ND	ND	109.5	(50.07, 168.92)	< 0.001	ND	ND	ND
Sleep disorders (yes vs. no)	198.9	(152.58, 245.23)	< 0.001	ND	ND	ND	-31.02	(-47.02, -15.02)	< 0.001

*Abbreviations* APD, antiparkinsonian drug; CI, confidence interval; MPR, medication possession ratio; ND, no data

^a^The unit of cost in this table is U.S. dollars. The U.S. dollar data were calculated based on the average exchange rate between the U.S. dollar and Chinese yuan in 2017

^b^Since some variables were dropped by the “backward method,” these blank spaces are filled by ND

### Sensitivity analysis

The mean (SD) PDC of all patients was 0.65 (0.26) (Table S3); PDC and MPR have similar determinants (Fig. S3) and similar effects on medical costs. Multivariate regression analysis results for the determinants of PDC indicated that a greater variety of APDs and all medications used and higher daily LED resulted in higher PDC, whereas digestive system diseases, epilepsy, or older age resulted in lower PDC (Fig. S4). The Gamma regression analysis results showed that a higher PDC resulted in higher APD costs (Table S4). For example, for an increase of 0.3 in PDC, APD costs increased by $36.49 (95% CI, $27.66–$45.33) per patient per year.

A total of 565, 574, and 573 patients had follow-up periods ≤ 290 d, 291–800 d, and ≥ 801 d, respectively (Table 1). The mean MPR/PDC values were lower in patients with a long follow-up period than in those with a short follow-up period (MPR, 0.67 vs. 0.78; PDC, 0.67 vs. 0.74). The proportion of patients with a high MPR/PDC ratio in the long-term follow-up group was lower than that in the short-term follow-up group (Fig. S5).

## Discussion

Electronic medical records of Chinese patients with PD demonstrated moderate medication adherence. Older age, concurrent digestive system diseases, and epilepsy were factors associated with poorer APD adherence, whereas an increased number of APDs or all medications, and higher daily LED resulted in improved adherence. The average annual total medical costs per patient were less than one-tenth the costs for patients with PD in developed countries. Higher APD adherence rates are associated with higher direct medical costs and older age is associated with increased medication expenses.

The results showed that comorbid epilepsy leads to poor medication adherence for patients with PD. In the cohort in the present study, the proportion of patients with PD with epilepsy at baseline was low (3.4%), corroborating data from other countries [28]. Epilepsy and dementia are comorbidities of PD, both of which increase the risk of cognitive impairment in patients [29, 30]. However, in the present study, when the two comorbidities were combined into a single variable, medication adherence was no longer affected. Moreover, the effect of epilepsy remained significant even after adjusting for patient age and other potential confounding factors. Prior research has demonstrated poor adherence to antiepileptic medication among patients with epilepsy [31]. Therefore, epilepsy may be a significant barrier to medication adherence in patients with PD. To the best of our knowledge, this is the first report of its kind within a real-world clinical setting. Consequently, further studies are required to establish definitive causal relationships. Clinicians and pharmacists should pay greater attention to patients with comorbid epilepsy during baseline assessment, as this factor can impact medication adherence, despite the relatively low prevalence of epilepsy among patients with PD.

The results of the present study indicated a negative association between age and medication adherence, which corroborates the results of prior research [19]. The results also demonstrated that concurrent digestive system diseases resulted in lower adherence rates to medication, similar to the findings of an earlier study [16]. In the present study, 20.9% of the patients with PD who had concurrent digestive system diseases at baseline had constipation and other types of esophagus/stomach/duodenum diseases. Constipation is a common adverse reaction of APDs [32], cited as the primary cause for the discontinuation of APDs by many patients [18]. Wallen et al. [33] proposed hypotheses regarding the role of the gut microbiome in PD pathogenesis, including the incomplete penetrance of PD susceptibility genes and potential triggers of pathology related to gut penetrance [33]. The findings, which highlight the factors responsible for reduced medication adherence, imply that patients with PD and comorbid digestive disorders may experience exacerbated symptoms after using APDs. In addition, the presence of comorbid digestive diseases in patients with PD was significantly associated with older age in the present study (Kolmogorov–Smirnov test, *p* < 0.001), emphasizing the importance of collaboration among gerontologists, gastroenterologists, and neurologists in the management of PD.

In the present study, the average annual total medical cost per patient with PD in China was estimated as $1,604.56, which was much lower than that of the patients with PD in the US ($23,041) and Sweden (€15,958) [34, 35]. The lower costs in China can be explained partially by low medication adherence rates. The cost of APDs in China is lower than in developed countries. The ratio of the median APD cost in China to that in the US, Australia, India, Canada, and the UK is 0.51 [36]. Especially after the Zero Markup Drug Policy for Public Hospitals was implemented in Beijing on April 1, 2017, the price of most APDs dropped [7], and the cost of managing PD has decreased [37].

Finally, the findings revealed that higher adherence to APDs is associated with the use of a larger number of medications and increased healthcare expenses, which is inconsistent with previous research findings [38]. Patients who use a variety of APDs or higher daily LED are often those who experience more severe PD symptoms and consequently adhere more closely to their physicians’ recommendations. Increased awareness of PD treatment may improve medication adherence [19]. Therefore, to improve medication adherence, patients with mild symptoms should also be considered when providing follow-up care, patient education, and other interventions. The fragmentation of China’s healthcare system can also explain such discrepant findings between the present study and prior research. Inherent in the Soviet model and the barefoot doctor movement [39], specialists in China are primarily concentrated in tertiary hospitals, and general practitioners are not well trained and lack capacity. Consequently, patients with PD are urged to seek treatment at tertiary hospitals to access initial or more effective medication regimens. In such hospitals, physicians often have no time or incentive to provide effective guidance. Moreover, they may not be aware of the medication status and associated costs for patients receiving care in other hospitals or regions. Data from the present study were collected from Peking University Third Hospital, which is a referral hospital located in Beijing, China. Patients without medical insurance in Beijing (34.9%) self-transferred to other provinces. For such reasons, policymakers should minimize regional differences in medical capacity to prevent patients from seeking healthcare across regions, and in turn, improve medication adherence.

The present study had several strengths. It was the first attempt to assess adherence to various types of APDs among Chinese patients with PD comprehensively. The authors attempted to include all potential comorbidities. To the best of our knowledge, this is the first report that considers epilepsy as a factor that is associated with poor APD medication adherence. The present study used electronic medical records of the patients treated at a 2,264-bed tertiary care medical center and teaching institution in Beijing. The records contained data from 3.94 to 4.22 million outpatient visits and 100,549–137,655 admissions, respectively, during the study period. China is one of the most rapidly aging countries globally; therefore, the present study on medication adherence for PD, conducted at a top tertiary hospital in the capital of China, not only has guiding significance for healthcare providers and policymakers in China but also has reference value for international peers.

The present study had some limitations. The data used covered only one medical center in Beijing, China, and the data collection period was only 32.5 months. The former may be the reason for the low inpatient admission rate of patients with PD during the study period and could explain the fact that out of 2,640 patients with PD diagnosis, only 1,712 patients were prescribed APDs more than twice. Electronic health records-pharmacy-linked data will facilitate a better generation of real-world evidence to more reliably evaluate APD medication adherence in future studies [40, 41]. The present study could not comprehensively present the medication status of patients with PD due to geographical and time limitations. Information regarding the stages of PD was not included in the data; therefore, the relationship between PD stages and medication adherence could not be evaluated. The data used in the present study only contained “prescribed use” information (APD filled data), without “actual use” information [42, 43], and did not contain the daily dosage of the prescription. Therefore, the supplied days of a single prescription could not be obtained by dividing the total dosage of the prescription by the daily dosage. Consequently, the calculations of the MPR, PDC, and daily LED were imprecise. Furthermore, APD data during hospitalization were not included because of the small size of inpatient admission data (Table S4), and gamma regression analysis was performed for APD costs, non-APD costs, and other outpatient costs. The impact of APD adherence on other costs (such as non-medical and invisible costs) and the economic burden on patients with PD requires further research. The second part of the sensitivity analysis (Fig. S5) indicated a trend of decreasing adherence with the extension of the follow-up time, which suggests that further studies on the changing regularity of medication adherence with an increase in PD treatment time can be significant and pioneering. This needs to be supported by longer follow-up data. The data do not provide information on patients’ income, marriage, and complexity of posology, which may affect PD patients’ adherence [18]. In addition, the Peking University Third Hospital introduced a collaborative pharmaceutical care service program in March 2017. Clinical pharmacists were engaged in the care of patients with PD, provided advice on how to use each medicine, and offered personalized explanations on request. Since doctors were not engaged in the interactions, the program may not affect patient diagnoses in the electronic medical records. However, the engagement of pharmacists may improve patients’ adherence [7]. Only 170 samples were available before the program, and there was a lack of adequate power (< 200) to detect the effect of the program. Future work is warranted to assess whether and how engaging clinical pharmacists in the care process could affect patients’ adherence, particularly for PD patients who used to have comorbidities.

Overall, the present study was a retrospective data analysis, which attempted to identify certain inferences related to medication adherence and aimed to guide future research. In future research, a structured approach (such as a framework based on timelines–events–objectives–sources) could be used [42] and comprehensive data (such as multi-center data, electronic health records-pharmacy-linked data, and databases containing treatment effects and outcomes) could be adopted to explore adherence measures closer to the real status, to identify other factors affecting APD adherence, such as collaborative pharmaceutical care service, different APD treatment regimens, other concurrent diseases, PD treatment time, and factors contributing to better treatment effects.

## Conclusion

APD adherence in a medical center in China was moderate, which was associated with patient age, comorbidities of digestive system diseases or epilepsy, total number of medications, and number and daily dosage of APDs. Healthcare providers should consider such factors when caring for patients with PD. Further studies are warranted to better understand the factors that influence APD adherence and the cost implications.

### Electronic supplementary material

Below is the link to the electronic supplementary material.

Supplementary Material 1

## Data Availability

The datasets generated and/or analyzed in the current study are available from the corresponding authors, ZMY and XLF, upon reasonable request.

## Abbreviations

APD: Antiparkinsonion drug
CI: Confidence interval
DOT: Duration of treatment
ICD: International Classification of Diseases
LED: Levodopa-equivalent doses
MD: Mean difference
MPR: Medication possession ratio
ND: No data
PD: Parkinson’s disease
PDC: Proportion of days covered
SD: Standard deviation
