# Supplementary material for: Medication adherence and costs of medical care among patients with Parkinson’s disease: an observational study using electronic medical records
Source: BMC Public Health. 2024 Apr 30;24:1202. doi: 10.1186/s12889-024-18431-y (PMC11061997; doi:10.1186/s12889-024-18431-y)
Supplement: Supplementary file 1 — Supplementary Material 1 [file 12889_2024_18431_MOESM1_ESM.pdf]

Supplementary Materials

Supplementary tables and figures

Table S1 The top 50 non-APDs according to the frequency of outpatient prescriptions

Table S2 Number of patients receiving antiparkinsonian monotherapy and their MPR/PDC values

Table S3 Characteristics and PDCs of the included patients with PD

Table S4 Gamma regression analysis of medical costs with PDC adherence measures

Fig. S1. Methods for measuring adherence

Fig. S2. Screening process of included patients

Fig. S3. Factors associated with adherence and the percentage of patients with low, medium, and high PDC in each subgroup

Fig. S4. Multiple linear regression for APD adherence using PDC

Fig. S5. Medication adherence of patients with different lengths of follow-up

Table S1 The top 50 non-APDs according to the frequency of outpatient prescriptions

Generic name		
Acarbose	Esomeprazole	Nicergoline
Amlodipine	Estazolam	Nifedipine
Aspirin	Finasteride	Olanzapine
Atorvastatin	Flupentixol and Melitracen	Omeprazole
Butylphthalide	Irbesartan	Paroxetine
Calcium Carbonate	Isosorbide Mononitrate	Rivastigmine
Citalopram	Lactulose	Rosuvastatin
Citicoline	Levamlodipine	Sertraline
Clopidogrel	Lorazepam	Sodium Valproate
Dihydroergotoxine	Mecobalamin	Sorbital
Donepezil	Memantine	TCM[a]
Duloxetine	Metformin	Zolpidem
Escitalopram	Mirtazapine	Zopiclone

Abbreviations: APD, antiparkinsonian drug; TCM, traditional Chinese medicines.

[a]TCM mainly refers to 12 types of Chinese patent medicines that improve constipation, gastrointestinal disorders, cardiocerebral circulation, and the common cold.

Table S2 Number of patients receiving antiparkinsonian monotherapy and their MPR/PDC values

Antiparkinsonian monotherapy	Patients, *n* /total *n* (%)	MPR, mean (SD)	PDC, mean (SD)
Subtotal	578/1712 (33.8)	0.59 (0.29)	0.57 (0.28)
Levodopa/benserazide	447/1712 (26.1)	0.59 (0.28)	0.57 (0.28)
Pramipexole	75/1712 (4.4)	0.52 (0.28)	0.50 (0.27)
Selegiline	20/1712 (1.2)	0.85 (0.18)	0.83 (0.20)
Piribedil	15/1712 (0.9)	0.49 (0.30)	0.48 (0.29)
Carbidopa/levodopa	7/1712 (0.4)	0.56 (0.29)	0.55 (0.30)
Amantadine	6/1712 (0.4)	0.78 (0.31)	0.68 (0.36)
Trihexyphenidyl	2/1712 (0.1)	0.20 (0.04)	0.20 (0.04)

Abbreviations: MPR, medication possession ratio; PDC, proportion of days covered; SD, standard deviation.

Table S3 Characteristics and PDCs of the included patients with PD

Characteristics	Patients, *n* (%)	PDC, Mean (SD)
Total sample	1712 (100)	0.65 (0.26)
**Baseline**		
**Sex**		
Female	736 (43.0)	0.64 (0.26)
Male	976 (57.0)	0.65 (0.26)
**Age range, y**		
≤44	33 (1.9)	0.70 (0.27)
45–64	529 (30.9)	0.67 (0.27)
65–74	404 (23.6)	0.66 (0.26)
75–84	616 (36.0)	0.63 (0.25)
≥85	130 (7.6)	0.59 (0.24)
**Number of all medications**		
1	153 (8.9)	0.50 (0.26)
2–5	998 (58.3)	0.64 (0.26)
6–10	493 (28.8)	0.71 (0.24)
11–36	68 (4.0)	0.69 (0.22)
**Concurrent diseases**		
**Neurological diseases**		
Depression: Yes	313 (18.3)	0.67 (0.25)
Depression: No	1399 (81.7)	0.64 (0.26)
Mental disorders: Yes	195 (11.4)	0.68 (0.25)
Mental disorders: No	1517 (88.6)	0.64 (0.26)
Dementia: Yes	286 (16.7)	0.63 (0.25)
Dementia: No	1426 (83.3)	0.65 (0.26)
Epilepsy: Yes	58 (3.4)	0.56 (0.31)
Epilepsy: No	1654 (96.6)	0.65 (0.26)
Sleep disorders: Yes	378 (22.1)	0.68 (0.23)
Sleep disorders: No	1334 (77.9)	0.64 (0.26)
**Circulatory system diseases: Yes**	710 (41.5)	0.64 (0.25)
**Circulatory system diseases: No**	1002 (58.5)	0.65 (0.26)
**Endocrine and metabolic diseases: Yes**	600 (35.0)	0.64 (0.26)
**Endocrine and metabolic diseases: No**	1112 (65.0)	0.65 (0.26)
**Digestive system diseases: Yes**	358 (20.9)	0.65 (0.24)
**Digestive system diseases: No**	1354 (79.1)	0.65 (0.26)
**Medical insurance: Yes**	1114 (65.1)	0.65 (0.26)
**Medical insurance: No**	598 (34.9)	0.64 (0.26)
**Follow-up period**		
**Follow-up days, d**		
≤290	565 (33.0)	0.74 (0.24)
291–800	574 (33.5)	0.54 (0.26)
≥801	573 (33.5)	0.67 (0.23)

Abbreviations: APD, antiparkinsonian drug; PD, Parkinson’s disease; PDC, proportion of days covered; SD, standard deviation.

(continued on next page)

Table S3 *(continued)*

Characteristics	Patients, *n* (%)	PDC, Mean (SD)
**Number of outpatient visits**		
2–6	815 (47.6)	0.61 (0.29)
7–12	383 (22.4)	0.60 (0.23)
13–18	235 (13.7)	0.67 (0.19)
19–24	137 (8.0)	0.77 (0.15)
≥25	142 (8.3)	0.86 (0.11)
**Inpatient episode: Yes**	115 (6.7)	0.65 (0.24)
**Inpatient episode: No**	1597 (93.3)	0.65 (0.26)
**Number of APDs**		
1	578 (33.8)	0.57 (0.28)
2	390 (22.8)	0.64 (0.26)
3	361 (21.1)	0.67 (0.23)
4	233 (13.6)	0.73 (0.21)
5–7	150 (8.8)	0.80 (0.18)
**Number of APD regimens**		
1	623 (36.4)	0.57 (0.28)
2	280 (16.4)	0.65 (0.27)
≥3	809 (47.3)	0.71 (0.22)
**Daily number of APDs**		
1	602 (35.2)	0.60 (0.29)
>1 and ≤2	744 (43.5)	0.71 (0.22)
>2 and ≤3	289 (16.9)	0.74 (0.21)
>3 and ≤5	77 (4.5)	0.85 (0.15)

Abbreviations: APD, antiparkinsonian drug; PD, Parkinson's disease; PDC, proportion of days covered; SD, standard deviation.

Table S4 Gamma regression analysis of medical costs with PDC adherence measures[a]

Variables	APD costs Estimate (95% CI)	Non-APD costs Estimate (95% CI)	Other outpatient costs Estimate (95% CI)	Inpatient admission costs Estimate (95% CI)	Inpatient admission costs[b] Estimate (95% CI)
Intercept	530.95 (500.43, 561.48) *[c]	199.99 (171.65, 228.32) *	106.67 (93.06, 120.29) *	5233.01 (3501.16, 6964.86) *	2145.23 (1062.41, 3228.05) *
Age (centered on 70 per 10 increase)	11.74 (5.12, 18.36) *	5.36 (4.41, 6.32) *	-18.64 (-27.5, -9.78) *	ND[d]	ND
Sex (male vs female)	ND	8.15 (6.58, 9.71) *	ND	ND	-1127.85 (-1747.52, -508.18) *
Medical insurance (yes vs. no)	-26.79 (-50.43, -3.14) *	3.87 (3.04, 4.7) *	ND	ND	ND
PDC (centered on 0.7 per 0.3 increase)	36.49 (27.66, 45.33) *	ND	ND	2192.52 (1073.11, 3311.93) *	ND
Number of APDs (centered on 2 per 1 increase)	322.52 (296.34, 348.69) *	-64.24 (-73.71, -54.77) *	ND	-1361.14 (-2079.04, -643.25) *	ND
Number of all medications (centered on 5 per 3 increase)	-19.31 (-23.47, -15.15) *	183.68 (155.11, 212.26) *	ND	849.73 (254.6, 1444.87) *	1696.8 (767.71, 2625.9) *
Circulatory diseases (yes vs. no)	ND	46.15 (18.98, 73.32) *	ND	ND	ND
Endocrine and metabolic diseases (yes vs. no)	-32.29 (-46.89, -17.7) *	65.36 (28.5, 102.21) *	ND	ND	ND
Digestive diseases (yes vs. no)	66.76 (37.42, 96.1) *	ND	ND	ND	-1130.56 (-1750.55, -510.56) *
Mental disorders (yes vs. no)	-36.18 (-53.84, -18.51) *	ND	32.21 (1.8, 62.62) *	-1736.68 (-2548.58, -924.77) *	ND
Dementia (yes vs. no)	ND	499.4 (356.1, 642.7) *	ND	-2524.18 (-4066.94, -981.42) *	-4981.13 (-7165.77, -2796.49) *
Depression (yes vs. no)	ND	109.5 (50.07, 168.92) *	ND	ND	1036.03 (-161.43, 2233.49) *
Sleep disorders (yes vs. no)	202.37 (156.41, 248.34) *	ND	-27.1 (-43.91, -10.3) *	ND	2189.49 (158.2, 4220.79) *
Number of inpatient admission	ND	ND	ND	ND	1779.82 (830.63, 2729.01) *

Abbreviations: APD, antiparkinsonian drug; CI, confidence interval; MPR, medication possession ratio; ND, no data; PDC, proportion of days covered.

[a]The unit of cost in this table is U.S. dollars. The U.S. dollar data were calculated based on the average exchange rate between the U.S. dollar and Chinese yuan in 2017.

[b]MPR as adherence measurement.

[c]* indicates P<0.05.

[d]Since some variables were dropped by the "backward method," these blank spaces are filled by ND.

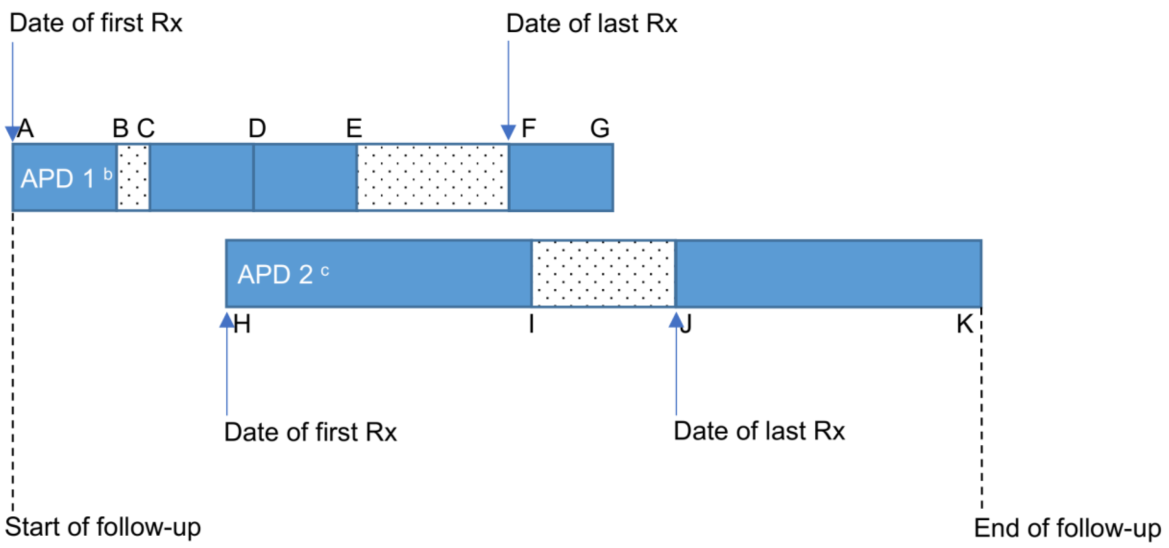

Adherence measure and calculation equation	Calculation methods
$\text{MPR} = \frac{1}{m}\sum_{i=1}^{m}\frac{n_i T_i}{\text{DOT}_i}$ [d]	$\text{MPR} = \frac{1}{2}\sum\left(\frac{4\times 30}{\text{days between A and G}} + \frac{2\times 90}{\text{days between H and K}}\right)$
$\text{PDC} = \frac{\text{days of at least one APDs applied}}{\text{DOT}}$	$\text{PDC} = \frac{\text{days between A and B} + \text{days between C and G} + \text{days between J and K}}{\text{days between A and K}}$

Fig. S1. Methods for measuring adherence[a]

[a]Hypothetical example represents a patient receiving pramipexole who initiated treatment with selegiline (1-medicine add-on).

[b]APD 1 indicates pramipexole.

[c]APD 2 indicates selegiline.

[d]The relevant parameters in the calculation equation are described as follows: "m" is the number of APDs prescribed, ranging from 1 to 7 in this case. "n_i" is the total times of prescription for APD i. "T_i" is the supplied days of a single prescription. For selegiline and amantadine, the supplied days of a single prescription (as "T" in this formula) is 90 days, while the other six APDs' are 30 days, according to the medical insurance regulations. The MPR was censored at one because some patients may have received the next prescription within 30 days.

Abbreviations: APD, antiparkinsonian drug; MPR, medication possession ratio; PDC, proportion of days covered.

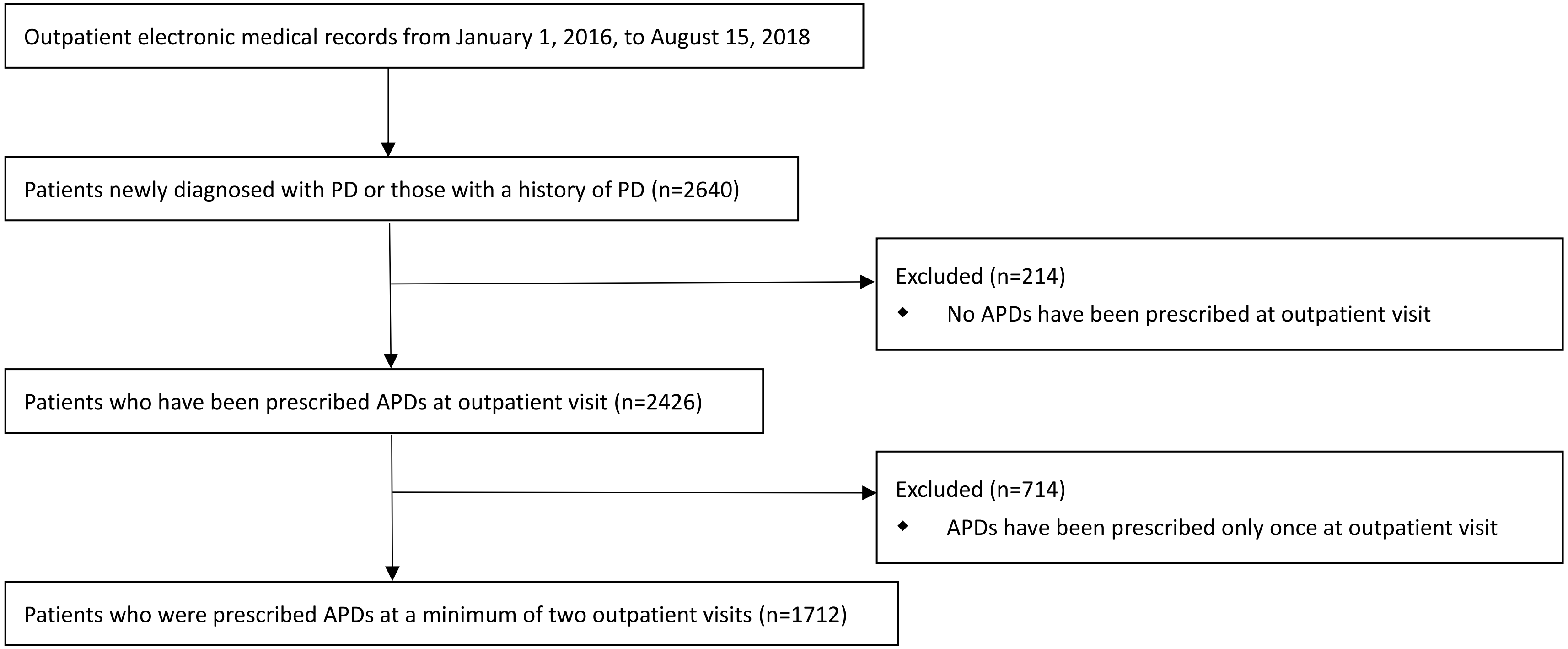

Fig. S2. Screening process of included patients

Abbreviations: APD, antiparkinsonian drug; PD, Parkinson’s disease.

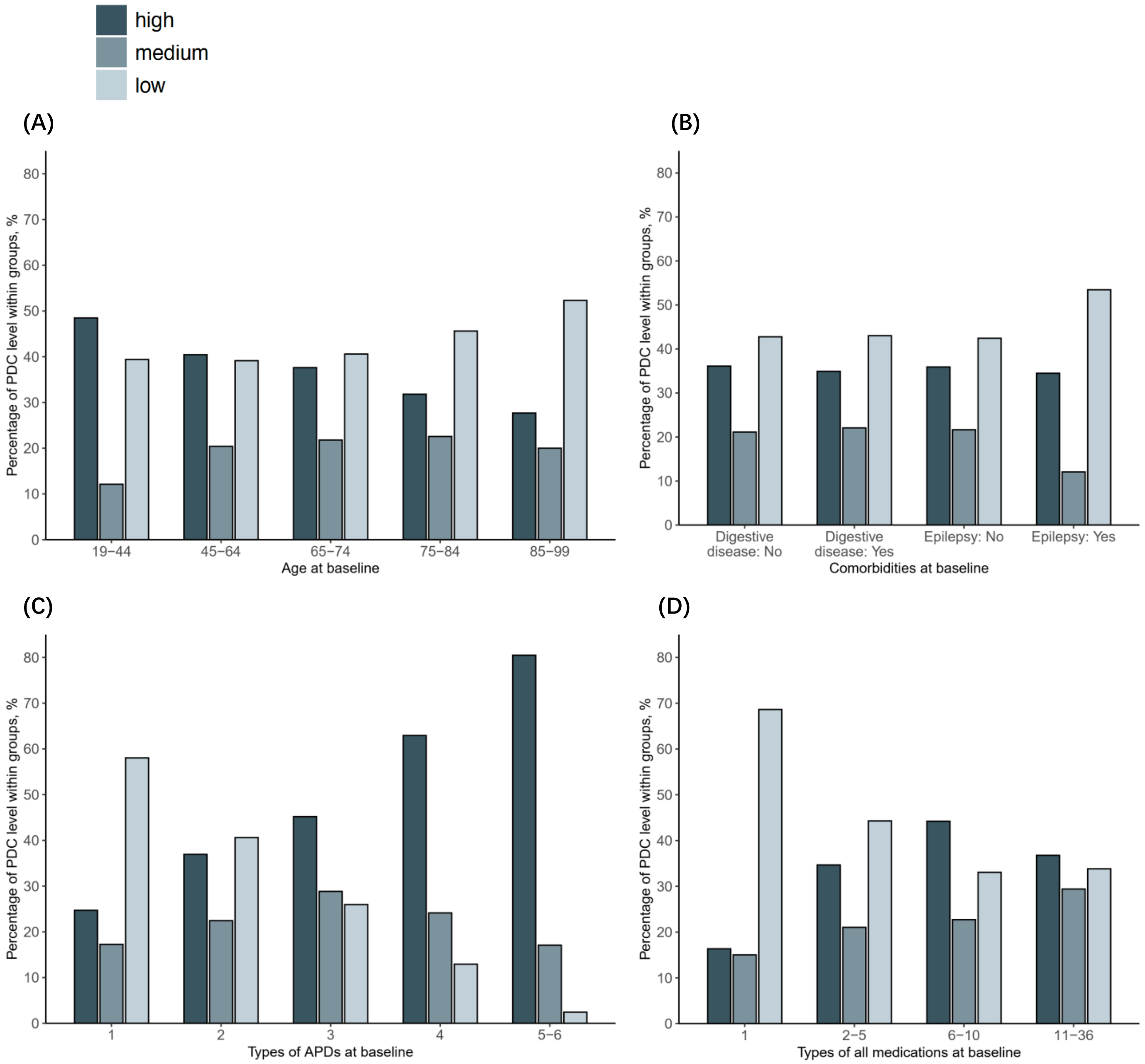

Fig. S3. Factors associated with adherence and the percentage of patients with low, medium, and high PDC in each subgroup

(A) Percentage of patients with low, medium, and high PDC in different age groups

(B) Percentage of patients with low, medium, and high PDC in the subgroup with comorbid digestive system disease or epilepsy

(C) Percentage of patients with low, medium, and high PDC in the subgroup with different types of APDs

(D) Percentage of patients with low, medium, and high PDC in the subgroup with different types of all medications

Abbreviations: APD, antiparkinsonian drug; PDC, proportion of days covered.

Variables	Mean difference (95% CI)
Epilepsy:<br>Yes vs. No	-0.07 (-0.13, -0.00)
Dementia:<br>Yes vs. No	-0.04 (-0.07, -0.01)
Digestive diseases:<br>Yes vs. No	-0.03 (-0.06, -0.00)
Age:<br>Centered on 70 per 10 increase	-0.02 (-0.03, -0.01)
Types of all medications:<br>Centered on 5 per 3 increase	0.02 (0.01, 0.04)
Daily LED:<br>Centered on .4 per .3 increase	0.03 (0.02, 0.05)
Mental disorders:<br>Yes vs. No	0.05 (0.01, 0.08)
Types of APDs:<br>Centered on 2 per 1 increase	0.07 (0.06, 0.08)

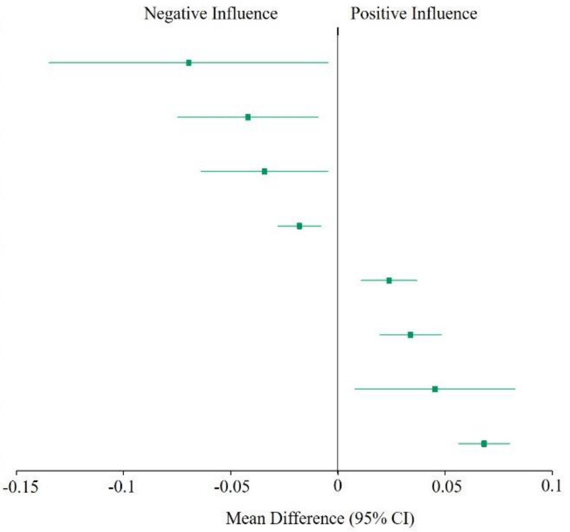

Fig. S4. Multiple linear regression for APD adherence using PDC[a]

[a]Stepwise regression was performed. Using the backward method, insignificant variables were dropped step-by-step. Hence, significant variables with a p-value less than 0.05 are selected in the end.

Abbreviations: APD, antiparkinsonian drug; CI, confidence interval; LED, levodopa-equivalent dose; PDC, proportion of days covered.

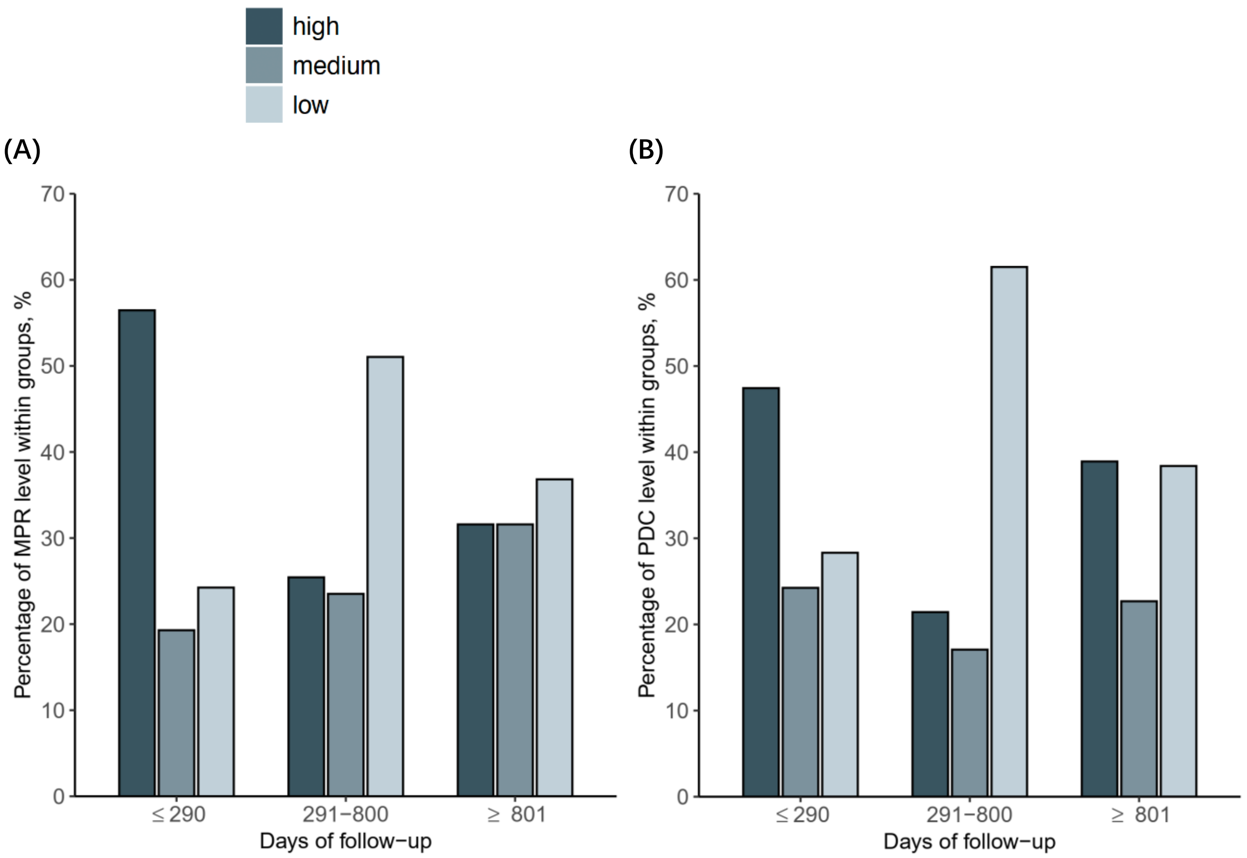

Fig. S5. Medication adherence of patients with different lengths of follow-up

(A) Percentage of patients with low, medium, and high MPR in different follow-up time subgroups

(B) Percentage of patients with low, medium, and high PDC in different follow-up time subgroups

Abbreviations: MPR, medication possession ratio; PDC, proportion of days covered.
